# Visceral fat correlates with insulin secretion and sensitivity independent of BMI and subcutaneous fat in Chinese with type 2 diabetes

**DOI:** 10.3389/fendo.2023.1144834

**Published:** 2023-02-27

**Authors:** Haishan Huang, Xiaobin Zheng, Xiaoming Wen, Jingyi Zhong, Yanting Zhou, Lingling Xu

**Affiliations:** ^1^ Department of Endocrinology, Shenzhen Hospital, Southern Medical University, Shenzhen, China; ^2^ The Third School of Clinical Medicine, Southern Medical University, Guangzhou, China

**Keywords:** insulin sensitivity, insulin secretion, abdominal obesity, visceral fat, type 2 diabetes mellitus

## Abstract

**Aim:**

Clinical heterogeneity exists in overall obesity and abdominal obesity in terms of insulin secretion and sensitivity. Further, the impact of visceral fat (VF) on the first- and second-phase insulin secretion (FPIS and SPIS) is controversial. We aim to investigate insulin secretion and sensitivity in Chinese patients with T2DM according to different BMI and VF levels.

**Methods:**

This study enrolled 300 participants. A dual bioelectrical impedance analyzer was used to assess the visceral and subcutaneous fat area (VFA and SFA). VF levels were categorized as normal or high, with the cutoff value of 100 cm^2^. FPIS and SPIS were evaluated by arginine stimulation test and standardized steamed bread meal tolerance test, respectively. β-cell function (HOMA2-β), insulin resistance (HOMA2-IR), and Gutt’s insulin sensitivity index (Gutt-ISI) were also calculated. Spearman’s correlation analysis and multivariate linear regression analysis were adopted for statistical analysis.

**Results:**

Participants were categorized into four groups: normal weight-normal VF, normal weight-high VF, overweight/obese-normal VF and overweight/obese-high VF. Multivariate linear regression showed that both VFA and SFA were correlated with FPIS, HOMA2-IR and Gutt-ISI after controlling for gender and diabetes duration. After further adjustment for BMI and VFA, some associations of SFA with insulin secretion and sensitivity disappeared. After adjustment for gender, diabetes duration, BMI and SFA, VFA was positively correlated with FPIS, SPIS and HOMA2-IR. Subjects with overweight/obese-high VF were more likely to have higher FPIS, HOMA2-IR and lower Gutt-ISI (all *p* < 0.05).

**Conclusion:**

VF affects both FPIS and SPIS, and worsens insulin sensitivity independent of BMI and subcutaneous fat in Chinese patients with T2DM.

**Clinical trial registration:**

http://www.chictr.org.cn, identifier ChiCTR2200062884.

## Introduction

Type 2 diabetes mellitus (T2DM) has become a major public health problem worldwide. The pathophysiology of T2DM is characterized by insulin resistance (IR) and β-cell dysfunction (1, 2). After disease onset, β-cell function progressively declines over time. Thus, exploring potential risk factors of β-cell dysfunction is important for preventing or delaying the development of diabetes (3, 4).

Obesity is a major risk factor for IR and T2DM (5). Although body mass index (BMI) is an internationally recognized index for diagnosing obesity, some studies have shown that obesity defined by BMI is remarkably heterogenous, and people with similar BMI do not have the same level of T2DM risk (6, 7). Abdominal obesity, specifically visceral adipose tissue (VAT), is associated with a greater risk of developing T2DM than peripheral obesity because expanded visceral fat stores affect insulin metabolism by releasing free fatty acids into the portal circulation, which may reduce the hepatic clearance of insulin, thus leading to IR and hyperinsulinemia (8). Further, Chinese have more visceral fat (VF) than Caucasians with the same BMI (9, 10). Therefore, it is significant to discover the differences of insulin secretion and sensitivity in Chinese with different types of obesity.

Measurement of VF accumulation is essential for the diagnosis of obesity. The visceral fat area (VFA) measured by dual bioelectrical impedance analysis (dual-BIA) is a simple and reliable method to estimate VF accumulation. Dual-BIA measures the bioelectrical impedance of the entire abdomen and its surface with a dual current path, which is considered better than the conventional BIA using only one current path and has high correlation with computed tomography (CT), a gold standard for VF accumulation (11, 12). To our knowledge, no population-based studies have examined the associations of β-cell function with VAT evaluated by dual-BIA.

The aim of this study was to examine the association of abdominal obesity assessed by dual-BIA with basal and post-load β-cell function, and clarify whether VAT and subcutaneous adipose tissue (SAT) have the same predictive effect on insulin secretion and sensitivity in Chinese patients with T2DM.

## Materials and methods

### Study population

This cross-sectional study recruited individuals hospitalized at the Department of Endocrinology of Shenzhen hospital, Southern Medical University, between August 2022 and November 2022. Inclusion criteria were Chinese participants who met the criteria of T2DM diagnosis based on the WHO consulting group (13); aged ≥ 18 years; BMI ≥ 18.5 kg/m^2^. Those with infectious diseases, cancer, or recent acute diabetic complications were excluded.

The study was approved by the Medical Ethics Committee, Shenzhen Hospital, Southern Medical University (NYSZYYEC202200017), and was registered at the Chinese Clinical Trials Registry (ChiCTR2200062884). All subjects signed informed consent before the investigation.

### Clinical measurements

Basic information, including age, gender, history of diabetes, history of diabetic complications and co-morbidities, drug use history, and other events in the exclusion criteria were collected. All participants underwent physical examination, which included measuring systolic and diastolic blood pressure (SBP and DBP), height (m), and weight (kg). BMI was calculated as weight (kg) divided by height (m) squared. Normal weight was defined as 18.5 ≤ BMI < 24 kg/m^2^, and overweight/obese was BMI ≥ 24 kg/m^2^ according to the Working Group on Obesity in China (WGOC) (2002) (14).

### Visceral fat area measurement by dual-BIA

VFA, along with subcutaneous fat area (SFA), was measured at the umbilical level by a dual bioelectrical impedance analyzer (Omron HDS-2000 DUALSCAN, Omron Healthcare Co, Kyoto, Japan), an equipment mainly designed to assess the abdominal fat area, as previously described (11, 15). Briefly, eight-point tactile electrode method was utilized according to the protocol. Resistance at five specific frequencies (1, 50, 250, 500 kHz, and 1 MHz) and reactance at three specific frequencies (5, 50 and 250 kHz) were measured to obtain the reading of VFA (cm^2^) and SFA (cm^2^) on the screen. The ratio of VFA and SFA (V/S ratio) was evaluated. All measurements were performed by the same experienced technician.

We used cutoff value of 100 cm^2^ in VFA to define visceral adiposity for both men and women (16). Thereafter, participants were categorized into four groups based on combinations of BMI and VF categories as follows: (1) normal weight-normal VF (18.5 kg/m^2^ BMI < 24 kg/m^2^ and VFA < 100 cm^2^), (2) normal weight-high VF (18.5 kg/m^2^ BMI < 24 kg/m^2^ and VFA ≥ 100 cm^2^), (3) overweight/obese-normal VF (BMI ≥ 24 kg/m^2^ and VFA <100 cm^2^), (4) overweight/obese-high VF (BMI ≥ 24 kg/m^2^ and VFA ≥ 100 cm^2^).

### Arginine stimulation test

AST was used to assess first-phase insulin secretion (FPIS) after overnight fasting for at least eight hours. After a baseline blood sample was collected, a 10% (wt/vol.) solution of arginine hydrochloride (5 g) (Shanghai Xinyi Jinzhu Pharmaceutical Co., Ltd., Shanghai, China) was injected intravenously within 30-45 s. Blood samples were obtained at 2, 4, and 6 min after injection (17). All anti-diabetic therapy was paused during the test.

### Standardized steamed bread meal tolerance test and biochemical measurements

The standardized steamed bread meal was made of 100 g flour, which contained carbohydrates approximately equivalent to 75 g glucose. The Chinese Islet Beta-Cell Function Collaborative Research Group showed that standardized steamed bread meal tolerance test (BMTT) was reproducible and was better tolerated when compared to oral glucose tolerance test (OGTT) to assess β-cell function in healthy subjects (18). Therefore, in China, BMTT is often used in clinical practice instead of OGTT to evaluate β-cell function in patients previously diagnosed with diabetes (19). Thus, we used BMTT to assess the second phase insulin secretion (SPIS).

Blood samples were collected in the morning under fasting conditions. Glycated hemoglobin (HbA1c), fasting plasma glucose (FPG), fasting insulin (FINS), fasting C-peptide (FCP), total cholesterol (TC), triglycerides (TG), high-density lipoprotein cholesterol (HDL-C), low-density lipoprotein cholesterol (LDL-C) and high-sensitivity C-reactive protein (Hs-CRP) were measured. Post-load blood samples were collected to assess 2 h plasma glucose (PG_2h_), 2 h insulin (INS_2h_) and 2 h C-peptide (CP_2h_) after the patients ate a 100 g steamed bread.

### Evaluation of insulin secretion and sensitivity

Since C-peptide response was equal to insulin response (20), the first and second-phase insulin release were separately calculated using the following formulate: FPIS = [(CP_2min_ + CP_4min_ + CP_6min_)/3 - CP_0min_]/[(PG_2min_ + PG_4min_ + PG_6min_)/3 - PG_0min_] and SPIS = (CP_2h_ – FCP)/(PG_2h_ – FPG) (21, 22). Basal β-cell function and IR were determined by employing updated Homeostasis Model Assessment (HOMA2) model of HOMA2-β and HOMA2-IR, which could be calculated by entering FPG and FCP into the HOMA Calculator software v2.2.3 (23). The postprandial insulin sensitivity index (ISI) was estimated according to the computation proposed by Gutt’s et al. (24). Therefore, we generated the following five indices: FPIS, SPIS, HOMA2-β, HOMA2-IR and Gutt-ISI.

### Statistical analyses

Data were analyzed using the SPSS software package (version 24.0; SPSS Inc, Chicago, IL, USA). Continuous variables were presented as means ± standard deviation (SD) for normal distribution or median with interquartile ranges for non-normal distribution. Categorical variables were presented as frequency (percentages). The Kolmogorov-Smirnov test was used to verify the normal distribution of continuous variables. The χ² test, one-way ANOVA or Kruskal-Wallis rank sum test were used to compare differences in categorical or continuous variables across the four groups, as appropriate. Relationships between abdominal fat distribution and β-cell function were analyzed using Spearman’s correlation analysis. All the covariates were tested for collinearity; the tolerance was > 0.1, and variance inflation factor did not > 5.0. Multivariate linear regression was used to assess the association of abdominal fat distribution with insulin secretion and sensitivity. A *p* value < 0.05 (two-sided) was considered as statistically significant.

## Results

The basic clinical characteristics of participants are summarized in **Table 1**. A total of 300 patients, 221 (73.67%) men and 79 (26.33%) women, with a mean age of 51.25 ± 11.96 years were included for data analysis. The median (25th, 75th percentile) VFA and SFA of the subjects were 100.00 (77.00, 127.00) cm^2^ and 171.00 (138.25, 211.00) cm^2^, respectively.

**Table 1:** Characteristics of study participants according to BMI and VF levels.

	Total (n=300)	Normal weight-normal VF (n = 99)	Normal weight-high VF (n = 31)	Overweight/obese-normal VF (n = 49)	Overweight/obese-high VF (n = 121)	*p* value
Age (years)	51.25 ± 11.96	53.04 ± 11.59	57.32 ± 10.30	51.86 ± 10.84	47.99 ± 12.26 **††	< 0.001
Male/female (%)	221/79 (73.67/26.33)	61/38 (61.62/38.38)	22/9 (70.97/29.03)	35/14 (71.43/28.57)	103/18 (85.12/14.88)*	0.001
BMI (kg/m^2^)	24.71 (22.68, 26.68)	22.27 (21.11, 23.23)	22.84 (21.30, 23.77)	25.15 (24.70, 26.17) ***†††	27.30 (25.62, 29.10) ***†††‡‡	< 0.001
Diabetes duration (years)	6.58 (1.00, 12.00)	8.00 (2.00, 14.00)	8.00 (2.00, 17.00)	8.00 (1.50, 14.50)	3.00 (0.67, 10.00) **	0.003
SBP (mmHg)	126.07 ± 17.20	120.81 ± 17.00	126.55 ± 19.54	124.59 ± 16.26	130.84 ± 15.93***	< 0.001
DBP (mmHg)	79.00 (72.25, 85.00)	75.00 (69.00, 80.00)	78.00 (71.00, 85.00)	77.00 (71.50, 84.00)	83.00 (76.00, 89.50) ***‡	< 0.001
Hs-CRP (mg/L)	1.31 (0.60, 3.13)	1.06 (0.40, 2.94)	1.30 (0.68, 2.76)	0.86 (0.29, 2.24)	1.91 (0.92, 3.73) **††‡‡‡	< 0.001
TG (mmol/L)	1.52 (1.04, 2.33)	1.23 (0.92, 2.00)	1.48 (0.92, 2.38)	1.51 (0.97, 2.17)	1.85 (1.27, 2.68) ***	< 0.001
TC (mmol/L)	4.41 (3.73, 5.09)	4.46 (3.52, 5.05)	4.26 (3.27, 4.90)	4.34 (3.77, 5.15)	4.42 (3.87, 5.11)	NS
LDL (mmol/L)	2.80 ± 0.98	2.86 ± 1.02	2.55 ± 0.88	2.81 ± 1.02	2.80 ± 0.94	NS
HDL (mmol/L)	1.06 (0.90, 1.26)	1.09 (0.95, 1.36)	1.12 (0.91, 1.37)	1.04 (0.86, 1.30)	0.99 (0.87, 1.17) **	0.006
HbA1c (%)	9.00 (7.30, 11.08)	9.50 (7.50, 11.20)	8.60 (7.20, 10.80)	8.70 (6.60, 11.25)	9.00 (7.70, 10.85)	NS
FPG (mmol/L)	7.31 (5.91, 9.38)	7.09 (5.87, 9.27)	7.27 (5.43, 8.30)	6.59 (5.24, 8.06)	8.12 (6.36, 9.97) ‡	0.010
PG_2h_ (mmol/L)	15.01 ± 4.73	15.24 ± 5.03	14.60 ± 4.62	14.40 ± 4.74	15.18 ± 4.52	NS
FCP (ng/mL)	2.01 (1.26, 2.78)	1.47 (1.02, 2.17)	2.08 (1.59, 2.52)	1.80 (1.10, 2.59)	2.54 (1.60, 3.31) ***‡‡	< 0.001
CP_2h_ (ng/mL)	4.87 (3.07, 7.21)	3.83 (2.46, 5.69)	4.58 (3.33, 7.92)	4.57 (3.33, 7.57)	6.15 (4.04, 8.27) ***	< 0.001
FINS (μU/mL)	6.88 (4.05, 11.08)	5.09 (3.22, 6.79)	6.85 (4.30, 9.81)	6.99 (3.62, 11.09)	10.21 (6.19, 14.89) ***†‡‡	< 0.001
INS_2h_ (μU/mL)	27.98 (14.83, 48.59)	21.81 (11.10, 34.44)	20.65 (14.02, 46.26)	28.55 (16.14, 51.11)	35.67 (21.78, 62.48) ***	< 0.001
VFA (cm/^2^)	100.00 (77.00, 127.00)	73.00 (52.00, 82.00)	119.00 (110.00, 130.00) ***	87.00 (71.50, 94.00) †††	129.00 (114.50, 153.50) ***†††‡‡‡	< 0.001
SFA (cm/^2^)	171.00 (138.25, 211.00)	133.00 (110.00, 153.00)	151.00 (130.00, 170.00)	174.00 (156.00, 193.50) ***	219.00 (186.50, 267.00) ***†††‡‡‡	< 0.001
V/S ratio	0.58 ± 0.18	0.50 ± 0.15	0.82 ± 0.16***	0.48 ± 0.10†††	0.61 ± 0.15***†††‡‡‡	< 0.001
FPIS	4.44 (2.22, 8.64)	3.22 (1.24, 5.23)	4.29 (2.52, 9.45)	4.05 (2.42, 6.68)	5.48 (3.05, 14.27) ***	< 0.001
SPIS	0.40 (0.19, 0.77)	0.32 (0.16, 0.63)	0.43 (0.19, 0.90)	0.42 (0.22, 1.00)	0.47 (0.24, 0.79) *	0.023
HOMA2-β (%)	57.05 (32.95, 97.35)	54.10 (29.30, 81.90)	57.60 (38.90, 129.90)	71.50 (33.15, 107.45)	58.90 (37.95, 98.05)	NS
HOMA2-IR (%)	1.67 (1.10, 2.30)	1.27 (0.88, 1.75)	1.78 (1.37, 2.10)	1.55 (0.90, 2.08)	2.13 (1.45, 2.83) ***‡‡	< 0.001
Gutt-ISI	45.78 (36.28, 59.52)	51.92 (41.37, 65.29)	48.43 (40.15, 65.50)	47.93 (35.08, 71.04)	39.77 (33.33, 51.59) ***†‡	< 0.001

Values are presented as mean ± SD for normally distributed data and median (25^th^, 75^th^ percentile) for non-normally distributed data, or n (%). p values for comparison between different groups were tested by χ² test for categorical data, one-way ANOVA for normally distributed values and Kruskal-Wallis test for nonparametric values. BMI, body mass index; SBP, systolic blood pressure; DBP, diastolic blood pressure; Hs-CRP, high-sensitivity C-reactive protein; TG, triglycerides; TC, total cholesterol; LDL, low-density lipoprotein; HDL, high-density lipoprotein; HbA1c, glycated hemoglobin; FPG, fasting plasma glucose; FCP, fasting C-peptide; FINS, fasting insulin; VFA, visceral fat area; SFA, subcutaneous fat area; FPIS, first phase insulin secretion; SPIS, second phase insulin secretion; IR, insulin resistance; ISI, insulin sensitivity index. NS, no significance.

*p <0.05, ** p <0.01, *** p <0.001, vs Normal weight-normal VF group;

†p <0.05, †† p <0.01, ††† p <0.001, vs Normal weight-high VF group;

‡p <0.05, ‡‡ p <0.01, ‡‡‡ p<0.001 vs Overweight/obese-normal VF group.

**Table 1** also shows the characteristics of subjects according to BMI and VFA levels. Among the 130 participants with normal weight, 99 had normal VF and 31 had high VF. Among the 170 participants who were overweight/obese, 49 had normal VF and 121 had high VF. The participants in the overweight/obese-high VF group were younger, had higher SBP, DBP, Hs-CRP, TG, FINS, INS_2h_, FCP, CP_2h_, FPIS, SPIS, HOMA2-IR, shorter diabetes duration, lower HDL and Gutt-ISI compared to the normal weight-normal VF group (all *p* < 0.05). In addition, the participants in the overweight/obese-high VF group had higher DBP, Hs-CRP, FPG, FCP, HOMA2-IR and lower Gutt-ISI than those with normal VF (all *p* < 0.05).

Spearman’s correlation analysis (**Table 2**) showed that BMI, VFA and SFA were positively correlated with FPIS (r = 0.302, 0.318, 0.294, all *p* < 0.001), SPIS (r = 0.152, 0.170 and 0.144, *p* = 0.008, 0.003 and 0.013), HOMA2-β (r = 0.119, 0.135 and 0.121, *p* = 0.039, 0.019 and 0.037), HOMA2-IR (r = 0.388, 0.418 and 0.432, all *p* < 0.001), and negatively correlated with Gutt-ISI (r = -0.271, -0.292 and -0.308, all *p* < 0.001), while there was no significant correlation between V/S ratio and the indexes of insulin secretion and sensitivity.

**Table 2:** Spearman’s correlation analysis for the association of obesity with insulin secretion and sensitivity.

	BMI		VFA		SFA		V/S ratio	
	r	*p*	r	*p*	r	*p*	r	*p*
FPIS	0.302	< 0.001	0.318	< 0.001	0.294	< 0.001	0.107	NS
SPIS	0.152	0.008	0.170	0.003	0.144	0.013	0.095	NS
HOMA2-β	0.119	0.039	0.135	0.019	0.121	0.037	0.087	NS
HOMA2-IR	0.388	<0.001	0.418	<0.001	0.432	<0.001	0.084	NS
Gutt-ISI	-0.271	<0.001	-0.292	<0.001	-0.308	<0.001	-0.051	NS

BMI, body mass index; VFA, visceral fat area; SFA, subcutaneous fat area; FPIS, first phase insulin secretion; SPIS, second phase insulin secretion; IR, insulin resistance; ISI, insulin sensitivity index. NS, no significance.

Multivariate linear regression was used to analyze the relationship between dependent (FPIS, SPIS, HOMA2-β, HOMA2-IR and Gutt-ISI) and predictor variables (VFA and SFA) (**Table 3**). After adjusted for gender and diabetes duration, either VFA or SFA was significantly and positively associated with FPIS (standard β = 0.229 and 0.149, *p* < 0.001 and *p* = 0.012), HOMA2-IR (standard β = 0.400 and 0.393, both *p* < 0.001) but negatively associated with Gutt-ISI (standard β = -0.199 and -0.158, *p* = 0.001 and *p* = 0.007). After further adjustment for BMI and VFA, the positive association of SFA with FPIS, HOMA2-IR and the inverse association of SFA with Gutt-ISI disappeared. After further adjustment of BMI and SFA, the positive association of VFA with FPIS (standard β = 0.225, *p* = 0.010), SPIS (standard β = 0.198, *p* = 0.024), HOMA2-IR (standard β = 0.211, *p* = 0.008) still remained significant but the negative association of VFA with Gutt-ISI disappeared.

**Table 3:** Multivariate linear regression analysis of abdominal fat distribution with insulin secretion and sensitivity.

Dependent variable	Predictor variables	Standard β (CI)	*p*
FPIS	VFA, adjusted for gender, diabetes duration	0.229 (0.091, 0.282)	< 0.001
	SFA, adjusted for gender, diabetes duration	0.149 (0.018, 0.139)	0.012
	VFA, adjusted for gender, diabetes duration, BMI and SFA	0.225 (0.045, 0.321)	0.010
	SFA, adjusted for gender, diabetes duration, BMI and VFA	-0.047 (-0.136, 0.087)	NS
SPIS	VFA, adjusted for gender, diabetes duration	0.066 (-0.005, 0.019)	NS
	SFA, adjusted for gender, diabetes duration	-0.020 (-0.009, 0.006)	NS
	VFA, adjusted for gender, diabetes duration, BMI and SFA	0.198 (0.003, 0.038)	0.024
	SFA, adjusted for gender, diabetes duration, BMI and VFA	0.004 (-0.014, 0.014)	NS
HOMA2-β	VFA, adjusted for gender, diabetes duration	0.122 (0.003, 0.298)	0.046
	SFA, adjusted for gender, diabetes duration	0.079 (-0.030, 0.156)	NS
	VFA, adjusted for gender, diabetes duration, BMI and SFA	0.085 (-0.107, 0.317)	NS
	SFA, adjusted for gender, diabetes duration, BMI and VFA	-0.129 (-0.274, 0.068)	NS
HOMA2-IR	VFA, adjusted for gender, diabetes duration	0.400 (0.008, 0.014)	< 0.001
	SFA, adjusted for gender, diabetes duration	0.393 (0.005, 0.009)	< 0.001
	VFA, adjusted for gender, diabetes duration, BMI and SFA	0.211 (0.001, 0.010)	0.008
	SFA, adjusted for gender, diabetes duration, BMI and VFA	0.178 (0.000, 0.006)	NS
Gutt-ISI	VFA, adjusted for gender, diabetes duration	-0.199 (-0.316, -0.081)	0.001
	SFA, adjusted for gender, diabetes duration	-0.158 (-0.176, -0.028)	0.007
	VFA, adjusted for gender, diabetes duration, BMI and SFA	-0.154 (-0.324, 0.017)	NS
	SFA, adjusted for gender, diabetes duration, BMI and VFA	-0.011 (-0.144, 0.130)	NS

BMI, body mass index; VFA, visceral fat area; SFA, subcutaneous fat area; FPIS, first phase insulin secretion; SPIS, second phase insulin secretion; IR, insulin resistance; ISI, insulin sensitivity index. NS, no significance.

To understand the indexes of insulin secretion and sensitivity associated with different obesity patterns, we developed multiple linear regression models (**Table 4**). Independent variables were normal weight-normal VF, normal weight-high VF, overweight/obese-normal VF, and overweight/obese-high VF. Dependent variables were FPIS, SPIS, HOMA2-β, HOMA2-IR and Gutt-ISI. After adjustment of gender and diabetes duration, FPIS, HOMA2-IR were higher and Gutt-ISI was lower in the overweight/obese-high VF group when compared to the normal weight-normal VF group and overweight/obese-normal VF group, respectively.

**Table 4:** Multivariate linear regression analysis of different types of obesity with insulin secretion and sensitivity.

	FPIS		SPIS		HOMA2-β		HOMA2-IR		Gutt-ISI	
	Standard β (CI)	*p*	Standard β (CI)	*p*	Standard β (CI)	*p*	Standard β (CI)	*p*	Standard β (CI)	*p*
Model 1										
Normal weight-normal VF (Reference)	0		0		0		0		0	
Normal weight-high VF	0.085 (-3.989, 22.384)	NS	0.023 (-1.368, 1.996)	NS	0.079 (-7.223, 33.207)	NS	0.075 (-0.148, 0.684)	NS	-0.033 (-20.420, 11.827)	NS
Overweight/obese-normal VF	0.029 (-8.604, 13.797)	NS	0.013 (-1.282, 1.575)	NS	0.121 (-.875, 33.468)	NS	0.059 (-0.180, 0.527)	NS	-0.022 (-16.124, 11.267)	NS
Overweight/obese-high VF	0.215 (5.369, 23.393)	0.002	0.027 (-0.921, 1.377)	NS	0.125 (-1.146, 26.487)	NS	0.369 (0.529, 1.097)	< 0.001	-0.227 (-29.616, -7.577)	0.001
Model 2										
Normal weight-normal VF	-0.037 (-13.797, 8.604)	NS	-0.017 (-1.575, 1.282)	NS	-0.154 (-33.468, 0.875)	NS	-0.075 (-0.527, 0.180)	NS	0.028 (-11.267, 16.124)	NS
Normal weight-high VF	0.061 (-8.072, 21.274)	NS	0.012 (-1.704, 2.039)	NS	-0.020 (-25.800, 19.191)	NS	0.027 (-0.368, 0.557)	NS	-0.014 (-19.809, 16.074)	NS
Overweight/obese-normal VF(Reference)	0		0		0		0		0	
Overweight/obese-high VF	0.176 (0.779, 22.790)	0.036	0.010 (-1.322, 1.485)	NS	-0.036 (-20.498, 13.246)	NS	0.290 (0.293, 0.987)	< 0.001	-0.197 (-29.625, -2.711)	0.019

VF, visceral fat; FPIS, first phase insulin secretion; SPIS, second phase insulin secretion; IR, insulin resistance; ISI, insulin sensitivity index; NS, no significance.

For all the models, FPIS, SPIS, HOMA2-β, HOMA2-IR and Gutt-ISI were used as dependent variables. Independent variables included gender, diabetes duration, normal weight-normal VF (as reference group in Model 1), Normal weight-high VF, Overweight/obesity-normal VF (as reference group in Model 2), and Overweight/obesity-high VF.

## Discussion

This study examined the cross-sectional associations of abdominal fat distribution with basal and post-load insulin secretion and sensitivity in Chinese patients with T2DM based on different BMI and VF levels. The results demonstrated that (1) after adjustment for gender, diabetes duration, BMI and VFA, some associations of SFA with insulin secretion and sensitivity indices disappeared. Of note, after adjustment of gender, diabetes duration, BMI and SFA, the association of VFA with FPIS, SPIS and HOMA2-IR still remained significant; (2) overweight/obese-high VF patients were more likely to have higher FPIS, HOMA2-IR and lower Gutt-ISI.

Our study found that VFA, rather than SFA, was associated with HOMA2-β, an index reflected basal insulin secretion; VFA was also correlated with post-load insulin secretion, including FPIS and SPIS, independent of SFA and BMI. The influence of obesity on insulin secretion is controversial. Kautzky-Willer et al. (25) found that there was no difference in the dynamic sensitivities to glucose of FPIS and SPIS as studied by the C-peptide minimal model. Bonadonna et al. (26) found an increase in both FPIS and SPIS as assessed by hyperglycemic clamp. Walton (27) et al. and Macor et al. (28) reported that an increased centrality of fat distribution was associated with an elevated SPIS rather than FPIS. Walton (27) et al. and Macor et al. (28) assessed FPIS by intravenous glucose tolerance test (IVGTT) while we assessed FPIS by AST in this study. Progressive impairment in the FPIS to glucose was evident with increasing severity of glucose intolerance; however, patients with T2DM may still have residual β-cell function in response to non-glucose stimulation (29), which may partly explain the association of VFA with FPIS in our study.

In addition to insulin secretion assessed by AST and BMTT, we also used HOMA2-IR and Gutt-ISI to assess insulin sensitivity. In general, HOMA2-IR are derived in the basal state and can therefore be considered to reflects basal or hepatic insulin sensitivity (30), whereas Gutt-ISI is a measure of post–glucose loading insulin resistance and represents both peripheral and hepatic insulin sensitivity, which have a higher correlation with the gold standard method for measuring insulin sensitivity: the euglycemic hyperinsulinemic clamp (31). The results that VFA correlated with HOMA2-IR independent to SFA and BMI suggested that VFA plays important roles in hepatic insulin sensitivity. The association of Gutt-ISI with VFA was disappeared when the confounders were added to SFA and BMI, which indicated that peripheral insulin sensitivity may also affected by SAT. Once SAT reaches its maximal expanding capacity, fatty acids redistribute ectopically in VAT and non-adipose tissues (32–34). Increased VAT leads to an increase in systemic release in resistin and possibly interleukins, and elevated circulating cytokines may play a role in the impairment of muscle insulin response (35). HOMA2-IR was higher and Gutt-ISI was lower in subjects of the overweight/obese-high VF group which confirmed that in subjects with higher VF, even though insulin secretion is higher, their β-cells could not compensate fully for decreased insulin sensitivity, thus leading to diabetes.

This study had several strengths. First, we assessed FPIS and SPIS by AST and BMTT, both of which generate a supraphysiologic insulin secretory response and are less technically demanding than methodologies such as hyperglycemic clamp. AST provides a measure of near-maximal insulin secretion (insulin secretory reserve) (36), while BMTT is easy to administer and is more suitable in β-cell function assessment than OGTT for subjects who have confirmed diabetes. Second, we assessed basal and postprandial insulin sensitivity by employing HOMA2 model and Gutt’s equation, respectively. Although hyperglycemic clamp is the gold standard to assess insulin sensitivity, it is technically challenging, while the indexes in our study are easy to calculate and are suitable for large sample size studies. Third, we divided participants into four groups based on BMI and VF levels, and found that even if subjects had same BMI levels, only those with high VF were associated with the indexes of insulin secretion and sensitivity.

This study had some limitations. First, the number of normal weight-high VF and overweight/obese-normal VF participants in this study was relatively limited; further, T2DM patients with the same BMI may have gender differences in the association of abdominal obesity with β-cell function though we did not find in the present study, a larger population study subgrouped by male and female is needed to confirm our results. Second, the levels of VAT and SAT were measured by dual-BIA rather than CT, which is a gold standard (37, 38). However, CT has problems of complexity, cost and X-ray exposure while dual-BIA, mainly designed to assess VFA and SFA, is simple and may have comparable effectiveness as CT (11, 12). Third, this was a single-center study, and the results might be applicable only to adults with T2DM in southern China.

In summary, the current study suggested that VAT affected basal and post-load insulin secretion and sensitivity. Hence, practitioners should not undermine the risk of IR and β-cell dysfunction in their patients entirely based on BMI, but consider fat distribution as well. For overweight/obese T2DM patients, especially those with VF accumulation, early intervention is needed to reduce VF, in order to delay β-cell dysfunction.

## Data availability statement

The original contributions presented in the study are included in the article/supplementary material. Further inquiries can be directed to the corresponding author.

## Ethics statement

The studies involving human participants were reviewed and approved by Medical Ethics Committee, Shenzhen Hospital, Southern Medical University (NYSZYYEC202200017). The patients/participants provided their written informed consent to participate in this study.

## Author contributions

HH conducted the statistical analyses and wrote the first draft of the manuscript. XZ and XW were involved in the interpretation of data. JZ and YZ contributed to the acquisition of data. LX designed the study and is the guarantor of this work. All authors contributed to the article and approved the submitted version.
